# Evaluation of pediatric residents’ attitudes toward ethical conflict: a cross-sectional study in Tehran, Iran

**Published:** 2017-02-28

**Authors:** Maliheh Kadivar, Marjan Mardani-Hamooleh, Shiva Shayestefar

**Affiliations:** 1 Professor, Division of Neonatology, Department of Pediatrics, Children’s Medical Center, Tehran University of Medical Sciences, Tehran, Iran.; 2 Assistant Professor, Department of Psychiatric Nursing, Iran University of Medical Sciences, Tehran, Iran.; 3 Pediatrician, Children’s Medical Center, Tehran University of Medical Sciences, Tehran, Iran.

**Keywords:** *Children*, *Education*, *Ethical conflict*, *Pediatrics*, *Resident*

## Abstract

Ethical conflicts are recognized as critical aspects in assessing competence in clinical communication. Moreover, pediatrics residents may face more problems, compared to other disciplines; due to the specific characteristics of the age group receiving services as well as the presence of their families. This study has been conducted with the aim of determining the attitude and perspective of pediatric residents toward ethical conflicts in the field of pediatrics. This descriptive, cross-sectional study was carried out on all residents of Tehran University of Medical Sciences (90 residents), selected through census method, in 2014. The data collection tool was a 32-item research-based questionnaire. Its validity and reliability were confirmed by the researchers and the medical faculty members. To analyze data, descriptive and inferential statistics were used. However, based on the results, lack of an advanced directive and written procedure for withdrawing life-sustaining treatment of an incompetent or critically-ill child (4.38 ± 0.80), lack of provision of sufficient information on obtaining informed consent (4.12 ± 1.10), and the absence of a legal written process for doing not resuscitate (DNR) orders (3.98 ± 0.95) were the most salient causes of ethical conflicts in pediatrics. Furthermore, in accordance with the linear regression analysis of demographic characteristics, there was a significant relationship (*P* = 0.04, r = 0.046) between residents’ year of education and attitude toward ethical conflict; however, this relationship was not observed in other demographic characteristics. Taking the priorities of ethical conflicts in pediatrics into account may help improve the designing of medical ethics education programs in hospitals for residents, thereby reducing the conflicts related to the issues of medical ethics.

## Introduction

Conflict is defined as an interpersonal or intrapersonal discord potentially causing serious harms (1). Our ethical experiences are riddled with a range of conflicts most human beings have gone through regardless of the precepts and code of ethics, so that they find themselves on the horns of dilemma and confusion in both discerning and practicing it. The concept of ethical conflicts was first introduced in Plato’s ideas and different views on this issue emerged in the post-platonic era (2). Moral conflicts are intertwined with our daily life and all human beings grapple with such situations (1). Although ethical conflicts are potentially possible across various areas and do occur within the field of medicine, they should be viewed according to the different medical disciplines in which they occur. Pediatric residents might encounter more problems than other disciplines in this area due to the specific characteristics of the age groups under their supervision along with the presence of the children’s families (3).

In the field of pediatrics, the refusal of the provision of treatment, order to discontinue resuscitation in the event of cardiorespiratory arrest, measures such as resuscitation of premature or newborn infants in severe conditions, children’s privacy, obtaining consent for treatment or intervention, parental disapproval of the necessary treatment, and child abuse or neglect by guardians are of the most important issues facing residents. These issues are the main causes of ethical conflicts in pediatric residents’ clinical practice (4). Another factor involved in the occurrence of ethical conflicts for residents is providing care for seriously ill children or at the end of life condition; the care of such children for the residents are associated with ongoing conflicts and further tensions (5). Furthermore, most pediatricians have experienced difficulties in interacting with critically ill children (4).

In this regard, the results of a study in England encompassing medical conflicts showed that decision-making processes for patients and communication between employees were the main ethical challenge in pediatric oncology from the perspective of professionals (6). In addition, a study was carried out with participation of the pediatric residents in the United States of America (USA) to expound their experiences in the Neonatal Intensive Care Unit (NICU) (5). Analysis of the participants’ experiences showed that the most common theme in this regard was conflict which further materialized in the relationships with families and other physicians. Furthermore, according to the residents, working in the NICU was fraught with distress because of their exposure to infant deaths (5). 

According to the results of a research conducted in Iran on the quality of medical ethics curriculum from the perspective of Iranian students, the program had an unfavorable condition (7). The results of another study in Iran suggested that one of the most significant clinical challenges cited by the medical students centered on issues related to informed consent (8). Moreover, physicians participating in an Iranian study believed that sometimes their efforts are not in line with their clinical commitments, have no benefit for their patients, and are considered futile (9). 

The trend of educating medical ethics in medical schools has had rapid progress. However, residency training has been focused more on the development of clinical skills, and most of the teaching hospitals have failed to either hold medical ethics education classes for the residents or dedicate far less hours to this end. Furthermore, the educational programs for residents do not cover the relationship between medical ethics and ethical decisions revolving around physicians and human values which are focused by both the teaching hospitals and the educational curricula (10). Therefore, given the importance of the moral concepts of pediatrics, a study was carried out in the USA to investigate the educational programs of pediatrics in terms of codes of ethics and professionalism policies by experts who supervised the program (5). It was found that although these programs pursue to implement ethical approaches; this issue does not represent a clear structure in the curriculum of pediatrics, and consequently, needs to be structurally revised (5). In this regard, the results of a study in Iran found that medical ethics rounds might be a helpful approach to teaching medical ethics to medical students (11).

Unfortunately, topics related to ethical conflicts of pediatricians are rarely argued in current medical ethics courses. In other words, although some researches have been performed to address the most common ethical challenges of medical ethics in Iran (9, 11-13), less attention has been paid to ethical conflicts in the pediatric field. Therefore, it seems that by identifying ethical conflicts in the field of pediatrics from the residents’ viewpoint, it is feasible to ameliorate the quality of treatment by residents in addition to their efforts toward upgrading the health-care service delivery trend by timely interventions, while reducing the conflicts.

## Method

In this cross-sectional study, the research population included all pediatric residents studying in 3 teaching hospitals affiliated to Tehran University of Medical Sciences (Tehran, Iran) (Imam Khomeini Hospital, Bahrami Hospital, and the Children’s Medical Center) for 9 months in 2014. Subjects were selected through census method (n = 90). The researchers, taking into account the ethical considerations, attempted to perform the study by gathering data using questionnaires filled out by the residents once obtaining permission from the Ethics Committee of Tehran University of Medical Sciences and the consent of residents.

The studied variables included demographic variables and residents’ attitudes toward ethical conflicts. The demographic variables studied included age, gender, marital status, years of residency, training hospital, and previous medical ethics course track records. First, the relevant literature, including theses, articles, and electronic databases (PubMed, Scopus, Science Direct, and ProQuest), were reviewed for an appropriate questionnaire. The used keywords were medical ethics, medical education, conflicts, challenges, and pediatric. The questionnaire used was made by the research group. For this purpose, during 3 sessions, the items were reviewed and revised using the views of 13 experts (10 pediatricians and 3 medical ethics professionals). Finally, the items were applied according to the latest comments and received the final approval of the panel of experts. Then, the final questionnaire was designed with 32 items. Each item was assessed based on a 5-point Likert scale ranging from 1 (strongly disagree) to 5 (strongly agree). The validity and reliability of this tool were justified by the researcher using content validity and test-retest methods. The content validity of this tool was measured by 10 faculty members of the department of pediatrics and department of medical ethics of Tehran University of Medical Sciences and their comments were applied. To verify the reliability of the mentioned tool, test-retest was conducted; 10 members of the sample group took the test 1 week apart. Results of the two tests were calculated using Pearson’s correlation coefficient to the accuracy of 0.78. 

The data were analyzed using descriptive (mean and standard deviation) and analytical (linear regression) statistics in SPSS software (version 16; SPSS Inc., Chicago, IL., USA). All *P* values < 0.05 were considered significance.

## Results

The findings related to the demographic characteristics of the participants are given in table 1. 

**Table 1:** The demographic data of residents

Demographic data	Number (%)
Age (year)	
25-35	58 (64.4)
Over 35	32 (35.6)
Gender	
Female	72 (80)
Male	18 (20)
Marital status	
Married	68 (75.5)
Single	22 (24.5)
Year of residency	
First	29 (32.6)
Second	30 (33.7)
Third	31 (34.4)
Teaching hospitals*	
A	17 (22.4)
B	41 (53.9)
C	18 (23.7)
Previous participation in medical ethics education	
Yes	72 (80)
No	18 (20)

* *The hospitals are presented as capital alphabetical letters for the purpose of confidentiality.*

Evaluation of the attitude of pediatrics residents toward moral conflicts indicated that lack of a formal and written process to withdraw life-saving treatments of seriously ill children toward the end of life (4.38 ± 0.80), lack of provision of adequate information in attaining informed consent (4.12 ± 1.10), and absence of a written procedure for do not resuscitate (DNR) orders for children (3.98 ± 0.95) are the primary causes of moral conflicts in the domain of pediatrics. The mean and standard deviations related to other priorities are presented in table 2. 

**Table 2:** Percentage, and mean of residents’ perspective toward ethical conflicts

Items	Strongly agree	Agree	Moderate	Disagree	Strongly disagree	Mean ± SD
Lack of a formal and written process to withdraw life-saving treatments of seriously ill child toward the end of life	24	22	20	18	16	4.38 ± 0.80
Lack of provision of ‎adequate information to attain informed consent	20	22	20	20	18	4.12 ± 1.10
Absence of a written procedure for DNR orders for children	20	33	22	13	12	3.98 ± 0.95
Lack of maintenance of respect and dignity of sick children and their family	20	31	22	12	15	3.96 ± 0.84
Violation of child’s privacy	18	30	24	16	12	3.92 ± 0.78
Violation of children’s rights as an educational subject	20	29	21	19	11	3.88 ± 1.20
Inappropriate method of conveying bad news to a sick child or parents	19	30	22	12	17	3.86 ± 0.62
Inappropriate professional behavior in communication between physicians and other health-care team members	20	28	23	16	13	3.84 ± 1.10
Conflict between hospital rules and professional ethics (code of ethics)	22	27	21	18	12	3.78 ± 0.68
Lack of respect for cultural and religious beliefs of sick children and their families	21	28	24	16	11	3.76 ± 0.46
Lack of pain control for the child	21	27	22	17	13	3.64 ± 0.82
Violation of rights of children as research subjects	22	24	26	11	17	3.61 ± 0.84
Inappropriate attitude toward decision-making conflicts between the sick child and his/her family	19	22	30	17	12	3.52 ± 0.68
Failure to establish the minimum physical requirements for continued mother-child intimacy	22	27	24	16	11	3.48 ± 0.72
Poor decision-making regarding colleagues’ misconduct	20	22	24	16	18	3.36 ± 0.44
Failure to establish parents’ right of staying with their sick child	18	21	29	20	12	3.23 ± 1.10
Inappropriate attitude toward sick child/family’s inappropriate request for futile treatment	18	22	30	17	13	3.18 ± 0.78
Inappropriate attitude toward or refusal of treatment by the sick child or his/her legal guardian	12	12	33	24	19	2.68 ± 0.66
Lack of suitable management of one’s medical error or that of colleagues	15	11	31	22	21	2.65 ± 0.76
Lack of appropriate action in cases of child abuse	12	11	30	27	20	2.62 ± 0.44
Implementation of physical limitations for sick children	11	16	28	24	21	2.56 ± 0.28
Inappropriate attitude toward the family’s misconduct toward their children	20	11	28	22	19	2.54 ± 0.38
Lack of a formal and written procedure for selection of ill children for allocation of limited resources	13	20	28	22	17	2.54 ± 0.42
Lack of integration of caring for sick children between different hospital units	13	15	29	21	22	2.52 ± 0.28
Breach of confidentiality of the child or parents	*9*	*22*	*27*	*22*	*20*	2.48 ± 0.66
Discrimination between sick children in terms of provision of services	13	18	27	18	20	2.46 ± 0.68
Euthanasia	13	18	27	20	22	2.46 ± 0.48
Lack of access of sick children and their families to an efficient system for handling complaints	11	15	24	30	20	2.42 ± 0.36
Intervention in areas lacking competency	11	17	27	24	21	2.36 ± 0.72
Lack of supervision over the safety of invasive procedures for patients	6	18	28	28	20	2.28 ± 0.48
Unnecessary referrals and measures for more financial benefits and personal gains	9	19	23	28	21	2.24 ± 0.38
Inappropriate appearance and clothing of medical staff	9	17	23	29	22	2.18 ± 0.46

*A P value < 0.05 was considered significant. *

*DNR: do not resuscitate, SD: standard deviation*

Furthermore, according to the linear regression analysis of demographic characteristics, there was a significant relationship between the residents’ years of education and their attitudes toward ethical conflict, but this relationship was not seen in any other demographic characteristics. The linear regression analysis results are presented in table 3.

**Table 3:** Results of linear regression test

Demographic data	Ethical conflicts	
	*P*	R
Age	0.68	0.089
Gender	0.59	0.059
Marital status	0.78	0.067
Year of residency*	0.04	0.046
Various teaching hospitals	0.88	0.025
Previous participation in medical ethics education	0.72	0.036

* Significant relationship

## Discussion

Assessing pediatrics residents’ attitudes toward ethical conflicts illustrated the lack of an enacted and written process to withdraw life-sustaining treatment toward the end of the child’s life, provision of adequate information for an informed consent, and an established procedure for DNR orders as the most important causative factors of ethical conflicts in the field of pediatrics according to the residents’ point of views.

By the same token, the findings of a study in the Netherlands showed that pediatricians face challenges regarding the issue of life-sustaining treatment for children, and therefore, they have focused on feeding medical information to their families (14). Another study was carried out in Canada to evaluate the attitudes of physicians toward the ethical challenges to life-sustaining treatments for children which, according to the findings, the most salient ethical challenge was related to fear of failure in the face of uncertainty (15).

A study was conducted with the participation of pediatricians in the Netherlands on the end-of-life decision-making for children (16). According to the results, all participants believed that it was best to discuss the matter with colleagues before talking to parents. In 50% of cases, experts declared that trying to expand parents’ awareness about their decision and seeking their permission are necessary. In 25% of cases, specialists commented that information should be presented to the parents without requiring their permission. In addition, 66 experts underlined the importance of pain management for children before their death. According to this study, the decision-making approach regarding the end-of-life in children is deeply influenced by the nature of decision and type of treatment (16).

However, it must be consisted that facing the death of children in the final stages of life is, by its very nature, a crucial factor shaping conflict in pediatricians. In this regard, the results of a research in the USA revealed that the factors playing a supportive role for pediatric residents in facing children’s death and helping them to reduce their perceived conflicts are teamwork and high quality end-of-life care provision (17).

The findings of this study and the results of other studies in this field suggest that life-sustaining treatment and DNR at the end stage of life are still challenging issues for which there is no legally-pronounced and written mandate. This issue is potentially harmful as far as ethical conflicts in pediatrics are concerned. Therefore, taking the fact into account that physicians encounter many conflicts at the final stage of children’s life, it is essential to use the right strategies of conflict resolution. 

In Iran, no approved clinical and ethical directive document and guideline exist to determine when and how DNR orders are assigned, and in some cases, this was communicated orally without the knowledge of the patient or his/her family. These ambiguities about DNR caused much controversy and confusion among the health staff about their course of action when managing a clinical situation. The fact of the matter is that making comments on the continuing treatment of patients in the terminal phase is a fundamental skill required by physicians and has given rise to scientific, ethical, and legal challenges.

Other results of this study showed that neglecting to provide enough information on obtaining informed consent is another effective factor in clinical conflicts in pediatrics. In this context, it should be acknowledged that although pediatricians play an important role in informing the patient about the consequences of disease (18), children’s eligibility in terms of giving informed consent depends on factors such as developmental age, the influences of parents and peers, quality of information provided, life experiences, and further medical decisions (19). It is clear that factors such as the impossibility of child's giving consent, and in some cases, surrogates’ incorrect decision-making (proxy decision-making), are issues that make appropriate ethical approaches toward sick children challenging. So that sometimes parents decision as to how to treat the child and follow treatment, from the standpoint of medical ethics, face the ethical challenge, and the accuracy of parents’ decision gets questionable. Frankly, there is currently no officially written plan designed for the Iranian families on decision-making in pediatrics in Iran, specifically on chronicle and life-threatening child diseases. Thus, in some cases, the child’s family is doubtful as to the appropriate decision. 

Results revealed that lack of respect for children and families, violation of children’s privacy and rights as an educational issue, and the improper process of breaking bad news to a sick child or parents are considered to constitute other important ethical conflicts in the field of pediatrics. These findings were also supported by a research carried out in Canada concerning this field of study (15). As believed by the researcher, breaking bad news is an integral part of the pediatricians’ duties This key element throws into stark relief the focus on ethical ingredients related to the profession for pediatricians. Therefore, regarding medical ethics and ethical requirements, on which relies the upholding of human values, and through taking advantage of a protectionist approach, the expression of truth is underlined to avoid aggravating the reality of ethical challenges.

As a matter of fact, health-care providers in the field of pediatrics, in most cases, do not involve the patient and his/her family in the treatment decision-making process. As a result, they fall short of the patient’s values, concerns, preferences, and barriers perceived by them to follow up on the treatment regimens, and in this way they in fact disrespect them. In contrast, addressing the issue of participative decision-making can boost satisfaction with care and quality of life (QOL) for the child and his/her family, and thus, help decrease the existing conflicts (20). In fact, based on clinical experiences, since the biggest concern of a sick child’s family is the suffering of their child, they expected experts and professionals to exert an attitude with maximum respect. Accordingly, it is essential that this humanitarian issue be addressed more profoundly by all pediatric subspecialists in the future.

On the other hand, educational programs in the health-care systems should be capable of training dexterous specialists. Therefore, patients need to be involved in medical education programs (10), yet the child’s privacy should be protected in this regard. Similarly, other researchers stated that despite the frequent access to patient records by medical students for educational purposes, broad questions are raised about ethical conflicts in this regard including maintaining patients’ data, issues related to autonomy, protecting privacy, and seeking their permission (21). As such, taking advantage of an ethical approach in establishing an educational communication with patients is necessary (22).

A study conducted in Sweden in collaboration with pediatricians in the field of cancer, illustrated that their biggest concern was the labels they allegedly received as messengers of life-threatening conditions and the breakers of bad news (23). This finding confirmed the results of the present study. According to this study, decision-making in difficult situations and breaking bad news are an integral part of pediatrics which emphasizes the importance of the ethical aspects related to the profession for pediatricians (23).

Other ethical conflicts considered important in this study were inappropriate professional behavior of physicians toward other health-care team members, and hospital rules and regulations which are in conflict with professional ethical principles. The results of a study in the USA indicated that negative factors related to professional behavior associated with the workplace that affect the work quality of pediatricians included the absence of professional autonomy, interpersonal challenges, poor communication among colleagues, non-supportive climate, and feeling of powerlessness (24). These findings emphasize the need to address the concept of professional ethics in pediatrics. One of the main concerns in the health services field is to respect values and ethical principles across inter-professional collaborations. To this end, a framework has been formulated in Iran which emphasizes the dire need of doctors in the field to observe the professional ethics principles, pay attention to the individual and social values, and manage ethical challenges in relation to a variety of intra-professional and inter-professional problems (25). 

In addition, lack of respect for cultural and religious beliefs of the sick children and their families was another ethical conflict of great importance from the viewpoint of the residents in this study. In a more realistic context, the process of both care and treatment of sick children is affected by cultural and social factors which are often misconceived by both the medical staff and families (26). Therefore, disdaining the cultural beliefs of the child and his/her family might give rise to ethical conflicts in the domain of pediatrics. This is a critical issue that must be addressed by the relevant specialists. 

Lack of complete and appropriate control and management of children’s pain, lack of respect for children’s rights as a research subject, dealing with a decision-making conflict between sick children and their families in an awkward way, and failure to provide suitable physical facilities for the continued presence of the mother in the hospital were other issues recognized as responsible for emergence of ethical conflict in this study.

Generally speaking, complementary education could be cited as one of the strategies that can assist residents in logically dealing with ethical conflicts. However, the findings of the study in Canada showed that medical ethics education is of very high value among residents, and familiarizing residents with moral conflicts during residency training is also important (27). In addition, moral counseling helps solve clinical challenges and results in better ethical clinical decision-making (28). It is clear that pediatric residents cannot avoid facing ethical conflicts, but they should be capable enough to prevent these conflicts through receiving appropriate training.

In this study, improper appearance and clothing of medical staff were the least important factor in the area of ethical conflicts. It seems that more studies should be conducted in this regard.

According to other findings, among the demographic characteristics, there was a significant relationship between year of residency and their attitude toward ethical conflict, but this relationship was not seen in any other demographic variable. This means that as residents proceeded to higher academic years, they may have developed a deeper understanding of the ethical conflicts related to their career. This finding may be explained in that the importance of ethical conflicts for residents has become more prominent with the passage of time along with further professional experiences.

This study had the following limitations. The pediatrics residents’ attitudes toward ethical conflicts regarding children were evaluated. The study findings, however, cannot be generalized to ethical conflicts in other specialized medical disciplines. Furthermore, another limitation of this study was that the perspective of pediatrics residents was evaluated in only one university of medical sciences, which is the largest university in the country with the highest population of residents in the field of pediatrics. Therefore, it is recommended that the opinions of residents from other medical specialties regarding ethical conflicts be evaluated in future studies to provide better comparisons in this regard. It is also suggested that a relevant national survey be conducted in Iran. On the other hand, comparison of the current research findings with those of other studies was limited due to the poverty of similar studies in Iran.

## Conclusion

Based on the results, formulation of clinical ethics standards and relevant educational methodology and their institutionalization into health centers, determination of strategic plans for clinical ethics education, identification of methods, the need to expand clinical ethics, and ultimately, its institutionalization into a comprehensive health-care system constitute the most important issues which must be considered for pediatrics residents’ ethical conflicts. This may help reduce the conflicts related to issues of medical ethics for them. In addition, it seems that formal medical ethical education should be incorporated in the formal curriculum of pediatrics in the country and the importance of these issues should be emphasized during the education of pediatrics residents.
